# Dynamics of Indian Ocean Slavery Revealed through Isotopic Data from the Colonial Era Cobern Street Burial Site, Cape Town, South Africa (1750-1827)

**DOI:** 10.1371/journal.pone.0157750

**Published:** 2016-06-16

**Authors:** Lisette M. Kootker, Linda Mbeki, Alan G. Morris, Henk Kars, Gareth R. Davies

**Affiliations:** 1 Geology & Geochemistry Cluster, Vrije Universiteit Amsterdam, Amsterdam, the Netherlands; 2 Research Institute for Culture, History and Heritage (CLUE+), Vrije Universiteit Amsterdam, Amsterdam, the Netherlands; 3 Department of Human Biology, Faculty of Health Sciences, University of Cape Town, Cape Town, South Africa; University of Otago, NEW ZEALAND

## Abstract

The Dutch East India Company (VOC) intended the Cape of Good Hope to be a refreshment stop for ships travelling between the Netherlands and its eastern colonies. The indigenous Khoisan, however, did not constitute an adequate workforce, therefore the VOC imported slaves from East Africa, Madagascar and Asia to expand the workforce. Cape Town became a cosmopolitan settlement with different categories of people, amongst them a non-European underclass that consisted of slaves, exiles, convicts and free-blacks. This study integrated new strontium isotope data with carbon and nitrogen isotope results from an 18^th^-19^th^ century burial ground at Cobern Street, Cape Town, to identify non-European forced migrants to the Cape. The aim of the study was to elucidate individual mobility patterns, the age at which the forced migration took place and, if possible, geographical provenance. Using three proxies, ^87^Sr/^86^Sr, δ^13^C_dentine_ and the presence of dental modifications, a majority (54.5%) of the individuals were found to be born non-locally. In addition, the ^87^Sr/^86^Sr data suggested that the non-locally born men came from more diverse geographic origins than the migrant women. Possible provenances were suggested for two individuals. These results contribute to an improved understanding of the dynamics of slave trading in the Indian Ocean world.

## Introduction

**Fig 1:**
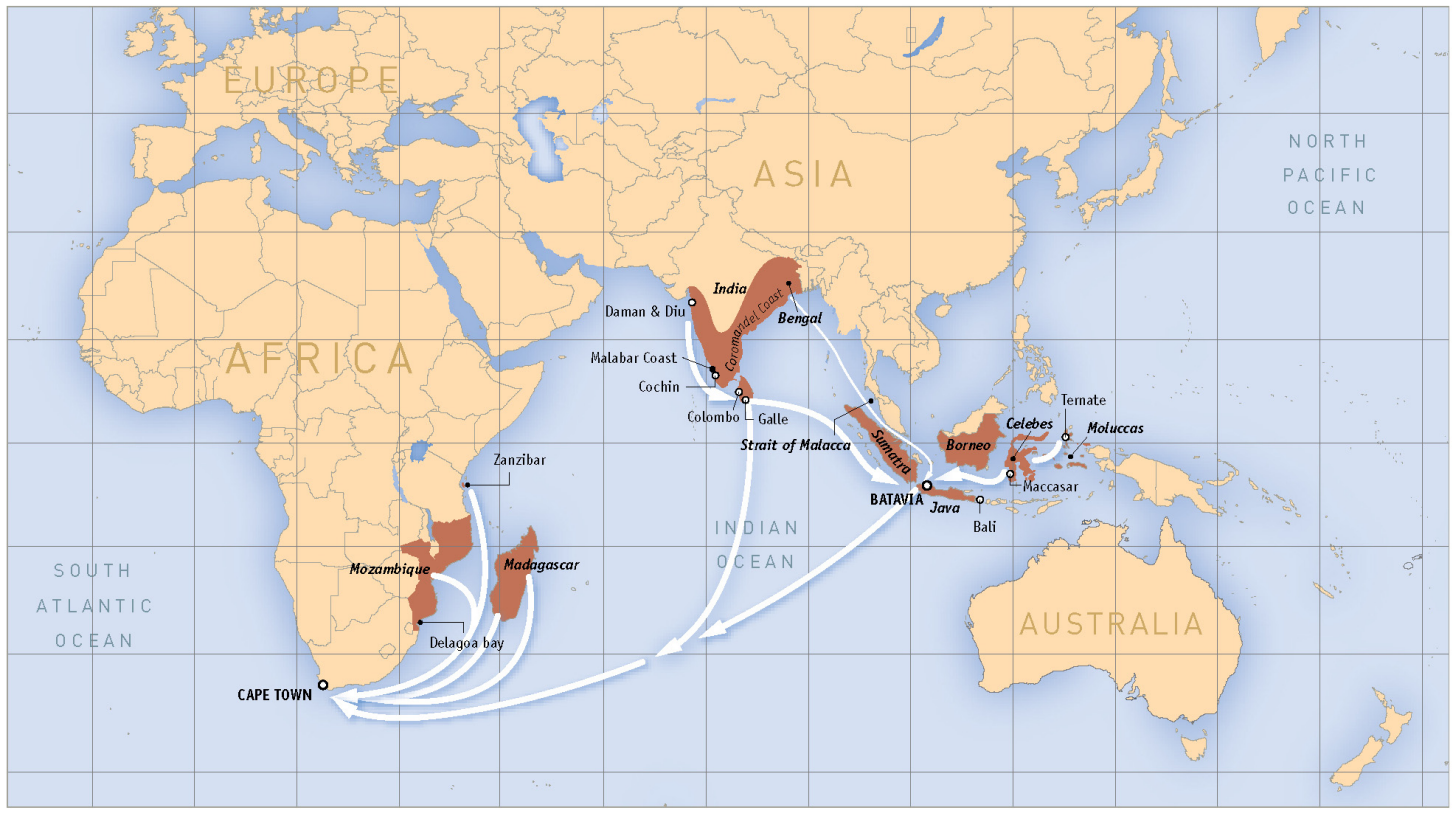
Indian Ocean slave trading routes. Map generated based on images provided by Iziko Museums and Frans Huijzenveld (Faculty of Humanities, department of Art and Culture, History, and Antiquity, Vrije Universiteit Amsterdam).

Between the years 1652 and 1795, the Dutch East India Company (VOC) governed the Cape Colony of present day South Africa. Save for a short-lived Batavian period (1803–1806), the British ruled the Cape from 1795 throughout the 19^th^-century. The VOC envisioned the Cape as a refreshment stop for company ships on their way to and from the East. Neither the VOC employees, nor the indigenous Khoisan produced adequate provisions to satisfy the demand of ships passing through the refreshment station. Thus, the VOC decided to import slaves to increase the available workforce. The VOC, however, could not rely on West Africa as a source of slaves because the Dutch West India Company had a monopoly on this trade [1]. In addition, the directors of the VOC, the Heren XVII or *the Gentlemen Seventeen*, did not support enslavement of indigenous people. Therefore, the VOC looked to eastern realms such as the east African coast, the middle South Asian circuit and the easternmost Asian circuit to supply the additional labour [2]. Records suggest that these three regions contributed approximately a quarter of the enslaved Cape population until 1808 when Britain outlawed the oceanic slave trade in its colonies. The remaining quarter of the Cape’s imported enslaved population originated from Madagascar [1]. These figures were not constant throughout this period as at any given time factors such as maritime conflicts and changing shipping patterns were at play [1]. Towards the end of the first Dutch administration, the colony relied less on imported Asian slaves and more on African slaves from the western Indian Ocean [3]. In their study of the 18^th^-century transoceanic informal trade in Asian slaves, Mbeki and van Rossum [4] reported that the majority of the enslaved transported by private persons to the Cape recorded toponyms from South Asia (65%) and the minority (33%) from the Indonesian archipelago (Fig 1). This study also suggested comparable numbers of men and women came from the Indonesian Archipelago, which is in contrast to the overwhelming preference for male slaves from south Asia. In general, men rather than women were favoured as slave labour at the Cape, particularly when agricultural production increased significantly in the hinterland. Of the enslaved that travelled on the Ceylon-Cape route, a large majority (89%) were from the Malabar Coast of India. The transportation patterns may have been affected by racial attitudes at the Cape that determined the kind of labour slaves were engaged in and the prices they fetched [4].

There have been several excellent reviews of the history of the Dutch Cape Colony and Indian Ocean slavery to which the reader is referred [1, 2, 5–11]. A current impediment to a full understanding of Indian Ocean World slave trades stems from a shortcoming in the historical record that often only provides information about slaves’ points of departure or sale as opposed to their places of origin [12]. Researchers of the Atlantic Ocean slave trade have begun to address this issue by adopting a biomolecular approach to investigate the life histories of enslaved people. Isotopic analyses have proven to be useful in elucidating mobility, (childhood) diet, manumission, and industrialisation in slave populations in for example the Caribbean [13, 14], the United States [15–17], and Mexico [18].

One of the major differences between the Indian Ocean and Atlantic Ocean slave trades is that the latter primarily involved a triangular movement of money, commodities, and people between Europe, Africa and the Americas. Enslaved Africans from West and Central Africa were transported to the Americas to labour on plantations. In contrast, the Cape was far more cosmopolitan than any of these nodes, with slaves coming from several slaving regions from the Indian Ocean basin. The complexity of the Indian Ocean slave system, however, has yet to be fully quantified (see [19] and [11] for historical research attempting this difficult task), and would benefit from further extensive biogeochemical and biomolecular studies to establish the provenance of slaves.

Pioneering work by Cox and Sealy [20] employing carbon, nitrogen, and strontium isotopes in conjunction with historical documents demonstrated the contribution that isotopic research could make to the study of Indian Ocean slavery. This was followed by the isotopic investigation of the subaltern population (non-Europeans including slaves and free-blacks, and possibly convicts and exiles) discovered at an informal burial ground at Cobern Street, Cape Town (*n* = 53) [21]. Using carbon and nitrogen isotopes Cox and colleagues [21] differentiated locally and non-locally born individuals based on dietary shifts. They proposed that a significant dietary shift in δ^13^C and/or δ^15^N between childhood (dentine samples) and later diet (cancellous bone samples) exceeding 2‰ can *provide strong confirmatory evidence for the presence of both first-generation slaves from Africa and the East and locally born people*. The study presented here compliments and refines their findings through a statistical reassessment of the δ^13^C data to establish more relevant background data. Moreover, strontium isotope analyses were performed on a selection of individuals from the same population (*n* = 35). The study was designed to elucidate individual mobility patterns, the age at which the forced migration took place and, if possible, geographical provenance. Ultimately, the results will contribute to an improved understanding of the dynamics of slave trading in the Indian Ocean in world history.

## Determination of Geological Origins through Isotope Analysis

The applicability of strontium isotopes to resolve environmental, ecological, archaeological, historical and forensic research questions has been illustrated by many scientific studies, *e*.*g*., [22–29]. The strontium isotope ratio ^87^Sr/^86^Sr serves as a powerful proxy to assign people and animals to specific geological areas [28, 30–33]. ^87^Sr is a radiogenic isotope, derived from the radioactive decay of ^87^Rb (t_1/2_ of 4.88 x 10^10^ years) [34]. The ^87^Sr content of a rock is a function of Rb content and the amount of time that has passed since its initial crystallisation [28, 35, 36]. Strontium passes from the geological bedrock into soil and is eventually taken up by vegetation [28, 35, 37, 38]. Since vegetation controls the ^87^Sr that enters the human and animal food chain [28, 39], it is noteworthy that the ^87^Sr/^86^Sr of vegetation differs slightly from the underlying geology, due to various soil-to-plant transfer factors, such as climate, fungi, root-depth and taxon [40, 41]. Moreover, in particular in coastal regions such as the Cape region, the effect of marine derived strontium on the deviation between floral ^87^Sr/^86^Sr ratios and geological ^87^Sr/^86^Sr ratios may be significant [14]. Crops in coastal regions may absorb marine derived strontium from rainwater, sea-spray and sea-splash, causing their ^87^Sr/^86^Sr ratio to shift towards a more marine signal (~0.7092 [14, 28, 35, 42, 43, 44]). Hence, the biologically available strontium may therefore deviate from the geological strontium isotope signature [14, 28, 45, 46].

Although mammals preferentially excrete ingested strontium via the kidneys and bile, a small proportion is retained in the body and incorporated into bone and dental enamel through diet. It then substitutes for calcium in the structure of hydroxyapatite (Ca_10_(PO_4_)_6_(OH)_2_), a calcium phosphate mineral [47]. Whereas bone constantly remodels during life, dental enamel develops during childhood and remains chemically unchanged in later life. The mineralisation age varies between dental elements, ranging from birth (first molars, M1) to approximately 16 years of age (third molars, M3) in permanent dentition [48–51]. Due to the difference in development, and age of incorporation of strontium, a difference in ^87^Sr/^86^Sr between bone and enamel could be interpreted as the result of migration in an individual’s lifetime. Diagenetic processes, however, lead to permanent alteration of the chemical and/or structural properties of bone and dentine [52]. Enamel is markedly less prone to diagenetic processes than dentine and bone, making it the preferred material for strontium isotope investigations [53–55]. Moreover, the study of different dental elements potentially allows the determination of the age at which migration took place provided it occurred in early life. Multi dental-elemental sampling, enabling inter-element comparison, therefore, offers a high-resolution manner to trace migratory patterns during early life (see Material and sampling strategy).

**Fig 2:**
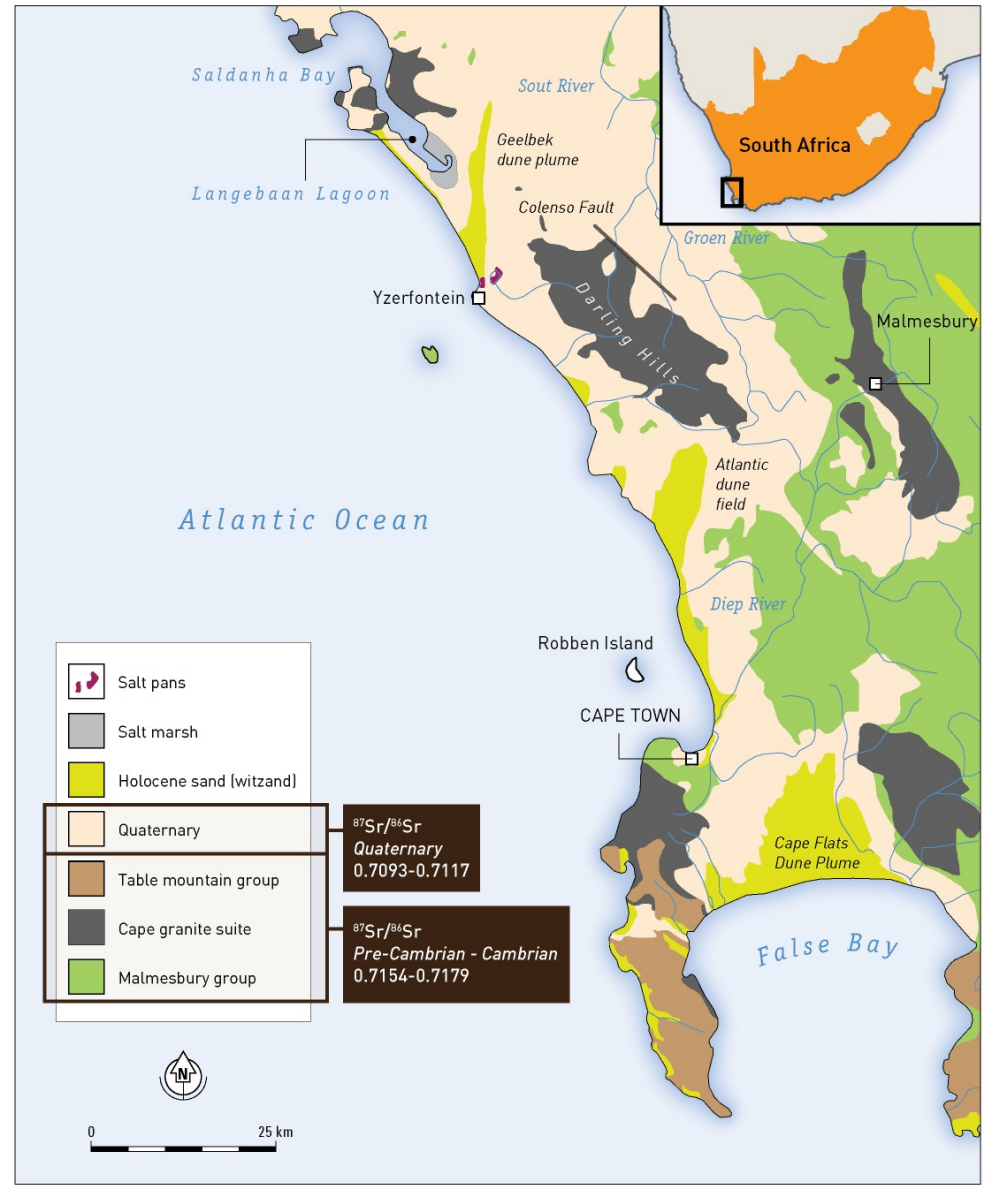
Schematic geological map of the southwestern Cape. Figure adapted from Compton *et al*. [70]. Strontium isotope data as published by Sealy *et al*. [69] and Balasse *et al*. [68].

The interpretation of ^87^Sr/^86^Sr ratios and the ultimate determination of geological provenance is highly dependent on available local or regional (bioavailable Sr) background data. Regional bioavailable ^87^Sr/^86^Sr distribution maps have been constructed for a few countries across the world, such as the United States [56], the United Kingdom [57, 58], France [59], the Netherlands [60], Germany [61, 62], Denmark [63] and Greece [64], while other archaeological studies in *e*.*g*. New Zealand and Thailand rely on the known geological data [65, 66]. A wide selection of papers report archaeological, geological or modern biosphere and faunal data from South Africa (see [67] for references), but to date no systematic study has been undertaken to map the spatial distribution of one of these proxies. Based on the bioavailable (fauna) data provided in Sealy *et al*. and Balasse *et al*. [68, 69], a schematic of the spatial distribution of ^87^Sr/^86^Sr in the Western Cape Province was generated (Fig 2).

An in-depth, but regionally applicable study by Maurer *et al*. emphasized the difficulties in determining the sources of strontium that enter the local food chain [46]. Moreover, they confirmed the offset in ^87^Sr/^86^Sr between geological, biosphere and faunal samples [38, 45]. Both factors hamper the accurate interpretation of the data in terms of provenancing, especially if only geological analyses are available to investigate origins. Nevertheless, the available data did provide some insight into the expected regional strontium ratios in the Cape region, and contributed to understanding the local strontium isotope signature.

## Material and Methods

The complete Cobern Street burial collection is curated at the Department of Human Biology, University of Cape Town. The collection is available for academic research. All necessary permits were obtained, which complied with all relevant regulations. Permission for sampling and analysis was granted by Heritage Western Cape (Ref.: 130129TS09) and an export permit for samples was obtained from the South African Heritage Resource Agency (Ref.: 9/2/018/0206. Permit ID: 219).

### Material and sampling strategy

In 1994, excavations revealed 63 intact primary burials belonging to the Cobern Street informal burial ground in Cape Town, which was used between circa 1750 and 1827 AD [21, 71]. The carbon and nitrogen isotope study by Cox *et al*. [21] was performed on 53 individuals, dating to the pre-colonial (<1652 AD) and colonial eras. The material and sampling strategy of the bone samples are provided in Cox [72], and will be summarised here. Cancellous bone samples were taken, preferably, from rib bones. If ribs were not available vertebrae were taken. A detailed overview of the samples taken for stable isotope analysis per individual is presented in Cox [72]. The rate of bone remodelling is influenced by bone type, bone element, age and a number of physiological and pathological factors [73]. Little quantitative are available, however, about collagen turnover rates [74]. It is known to vary between 2% and 4%/yr in femoral cortical bone in adults, with much higher rates up to even 100% reached during growth [75]. The turnover rate of trabecular bone, and in particular that or ribs and vertebrae, is faster than cortical bone and collagen (on average 10%/yr in adults: [76]). The carbon isotope data published by Cox *et al*. [21] and reassessed in this study therefore provide information on the dietary intake of the last circa 5 years of life. In contrast to bone, there is no significant turnover or replacement of dentine [77]. As a result, the δ^13^C value of dentine is directly related to the dietary intake of carbon during the time of root formation, which is element dependant. In permanent dentition, the initiation of root formation (developmental stage R_i_: [78]) can start as early as four years of age and finish as late as the early twenties (apex completed: A_c_). The selected dental elements and the interpretational consequences of that selection are specifically discussed in Cox [72].

For this research, a complete overlap with the Cox *et al*. [21] dataset could not be achieved due to insufficient sample material. To enable meaningful comparison, and depending on availability, enamel was sampled from 35 individuals dating from the colonial era. Where possible, first molars were selected for single-elemental analysis to distinguish locally and non-locally born individuals.

Multi dental-elemental analysis was performed on preferably first, second and third molars of a subset of individuals (n = 17) to further investigate individual migration events in early life (Table 1). Slight variations in mineralisation age are observed between European, Asian and African populations [49, 79]. Despite the fact that persons of Asian ancestry were present in the colonial Cape [1], in this paper the tooth crown initiation (C_i_) and completion (Cr_c_) times for Southern African populations published by Reid and Dean [49] are used for reference (Table 2). First evidence of calcification of the crown (C_i_) of the first molar is observed at birth. The last state of dental enamel formation, crown completion (Cr_c_), finishes around the age of three. The observed ^87^Sr/^86^Sr ratio of this element, therefore, reflects the dietary ^87^Sr/^86^Sr intake during the first three years of life. Since the M1 mineralises while the infant is likely to be breastfed, the mother’s dietary ^87^Sr/^86^Sr intake will be (partly) reflected in the children’s deciduous teeth and molars, which mineralise in the womb (*in utero*), and the first permanent molar. The second and third permanent molars mineralised between the ages of circa 3 and 6, and 8 and 16 years respectively.

**Table 1:** Osteological data and selected teeth for strontium isotope analysis from 35 individuals from the Cobern Street informal burial site, Cape Town, dating to the colonial period.

Burial type	Burial #	UCT #	Age (yr)	Sex	Dental modifications?	dI1 or dI2	I2	PM1	M1	M2	M3
B	3	460	20	M	-	-	-	-	16	-	-
B	4	458.1	17–18	F	-	-	-	-	16	-	-
B	10	498	35–40	F	-	-	-	-	26	-	-
B	12	500	35–40	M	-	-	-	-	46	-	-
B	13	501	30–40	M	-	-	-	-	46	-	-
B	14	502	45–55	F	-	-	-	-	46	47	48
B	15	504	25	M	-	-	-	-	46	-	-
B	18	508	>50	F	-	-	-	-	26	47	18
B	20A	510	25–30	M	The maxillary central incisors were chipped mesially at the midline, and the lateral maxillary incisors were chipped distally to form inverted 'V' shapes	-	-	-	16	17	18
B	20B	511	16	F	The maxillary central incisors were chipped mesially at the midline, and the lateral maxillary incisors were chipped distally to form inverted 'V' shapes	-	-	-	46	47	38
B	20C	512	1,5–2	?	-	71/2 or 81/2	-	-	-	-	-
B	21	514	25–35	F	-	-	-	-	46	47	38
B	23	516	17–19	F	-	-	-	-	36	37	38
B	24	517	20–25	M	-	-	-	-	36	-	-
B	25	518	35–40	M	-	-	-	-	46	-	-
B	27B	521	40–15	M	-	-	-	-	26	-	-
B	28	522	50	F	-	-	-	-	46	-	-
C	32	526	50–60	M	-	-	-	-	46	17	48
B	34	528	14–15	F	-	-	-	-	46	-	-
B	40	535	12	F	The maxillary central incisors were chipped mesially at the midline	-	-	-	46	-	-
B	41	536	35–50	M	-	-	-	-	36	-	-
B	42A	539	40–50	M	-	-	-	-	16	-	-
B	42B	540	40–50	F	-	-	-	-	26	-	-
B	44	542	40–50	F	-	-	-	-	46	47	48
B	45	543	50+	M	-	-	-	-	-	37	-
B	46	544	35–50	F	-	-	-	-	16	-	-
B	47	545	30–40	M	-	-	-	-	46	-	-
B	49	547	30–35	M	-	-	-	-	26	47	-
B	50	548	35–50	M	The central maxillary incisors were chipped distally, and the lateral maxillary incisors were chipped mesially.	-	-	-	46	47	48
B	51	549	35–40	M	-	-	-	-	26	-	-
B	52	550	25–35	F	The chipping of the maxillary central and lateral incisors to points.	-	-	-	46	47	48
B	53	551	35–40	M	-	-	-	44	-	-	-
B	54	552	30–35	M	-	-	-	-	46	47	48
B	56	554	35	M	-	-	-	-	46	-	-
C	57	555	20–30	F	-	-	-	-	36	47	18
B	58	556	35–40	F	-	-	32	-	-	17	48
C	59	557	40	M	-	-	-	-	36	37	18
B	60	558	30	F	The central and lateral maxillary incisors are sharpened to a points, by chipping the incisors both mesially and distally.	-	-	-	36	37	18
B	61	559	35	M	-	-	-	-	46	-	-
C	65	563	22–25	F	-	-	-	-	26	27	18

Key: Archaeological and osteological data from [71], dental modofication data from [71, 72]. Burial type refers to supine/Christian style (B) or facing Signal Hill/Muslim style (C). UCT number refers to the accession number. Dental element notation conforms to *Fédération Dentaire Internationale* (syntax: <quadrant code><tooth code>. Details presented in Table 2). dI1 of dI2: deciduous central or lateral incisor. I2: permanent lateral incisor. PM1: first premolar. M1: permanent first molar. M2: permanent second molar. M3: third molar.

**Table 2:** Chronology of human dentition in South African populations. Average crown formation times from [49].

		FDI notation		Average crown formation times	
Dentition	Tooth	Right quadrant	Left quadrant	Initiation (C_i_)	Completion (Cr_c_)
Permanent maxillary teeth	Central incisor	11	21	4 mon.	4.1 yr.
	Lateral incisor	12	22	12.5 mon.	4.8 yr.
	Canine	13	23	9 mon.	4.8 yr.
	First premolar	14	24	1.5 yr.*	6 yr.*
	Second premolar	15	25	2 yr.*	7 yr.*
	First molar	16	26	Birth	2.9 yr.
	Second molar	17	27	3 yr.	6.4 yr.
	Third molar	18	28	8 yr.	11.3–16 yr.**
Permanent mandibular teeth	Central incisor	41	31	3 mon.	3.4 yr.
	Lateral incisor	42	32	5 mon.	3.8 yr.
	Canine	43	33	6.5 mon.	5.2 yr.
	First premolar	44	34	1.75 yr.*	6 yr.*
	Second premolar	45	35	2.25 yr.*	7 yr.*
	First molar	46	36	Birth	3 yr.
	Second molar	47	37	3 yr.	6.2 yr.
	Third molar	48	38	8 yr.	11.2–16 yr.**

Key: C_i_: FDI notation: a two-digit system (ISO 3950) developed by the Feédeération Dentaire Internationale (FDI) to associate information to a specific tooth. Syntax: <quadrant code><tooth code>. Deciduous teeth quadrant codes start with 5 (left quadrant maxilla), followed by 6 (right quadrant maxilla), 7 (right quadrant mandible), and 8 (left quadrant mandible); C_i_: cusp initiated; Cr_c_: crown completed (developmental stages conform to Moorrees [78]);

*: no data published by Reid and Dean [49]—formation ages adapted from Nelson and Ash [50];

**: formation age extended to 16 due to observed inconsistencies and variations in literature with regards to M3 crown formation times [50, 89].

To test the hypothesis that a significant dietary shift is indicative of migration [21], multi-dental elemental sampling was performed on eight individuals who were assumed to represent migrants based on the presence of such a significant dietary shift in δ^13^C (burials 14, 18, 21, 32, 49, 54, 57, and 58). An additional five individuals (burials 20A, 20B, 50, 52, and 60) were selected for multi-dental elemental sampling based on the presence of dental modifications. Intentional dental modifications present in the Cobern Street collection (see [71, 72] for a summary of the unpublished data by Morris and Phillips [80]. Illustrative images are provided in Manyaapelo [81]) were not a tradition at the Cape at any time, and are therefore indicative of foreign origins [81]. Four individuals were selected for multi-dental elemental analysis based on the absence of such a significant dietary shift (random selection: burials 23, 44, 59, and 65). It is noteworthy that individuals 44, 49, and 65 showed a shift in δ^15^N exceeding 2.0‰. In contrast to Cox *et al*. [21], however, individuals with significant isotopic differences between δ^15^N_dentine_ and δ^15^N_cancellous_ were not assigned as migrants. This is due to the fact that there are multiple contributing factors that influence δ^15^N, such as sea spray, precipitation, fertilisers, disease, malnutrition and water stress [82–88]. Eighteen individuals, who were assumed to be locally born, or second- or subsequent generation slaves, were selected for single-elemental analysis. The individuals sampled for isotopic analysis represented two distinct patterns of burial placements, defined as type B (supine burial, Christian style) and type C (buried on their right side, Muslim style).

### Analytical methods

Osteological methods and analytical details of the carbon isotope analyses were provided elsewhere [71, 72]. For the strontium isotope analysis, dental enamel samples were obtained at the Department of Human Biology, University of Cape Town. Mechanical cleaning was performed on the enamel using an acid-leached diamond-tipped drill bit to expose a dull white surface visually unaffected by diagenetic alterations. Approximately 1–3 milligrams of enamel powder were collected from the buccal or lingual surface, depending on the state of preservation of the dental element, sealed in acid-cleaned polyethylene Eppendorf centrifuge tubes and transported to the class 100 clean laboratory at the Vrije Universiteit Amsterdam. The samples were leached with 0.1N acetic acid (CH_3_CO_2_H) to remove labile diagenetic strontium [55], and eventually dissolved in 3.0N nitric acid (HNO_3_). Strontium was isolated by ion exchange chromatography using Sr-Resin (EIChroM) and collected in acid-leached Teflon vials (Savillex). Blanks were spiked with ^84^Sr. All samples were nitrated twice with concentrated HNO_3_ before isotopic analysis.

The samples were loaded on single annealed rhenium filaments with TaCl_5_. The measurements were performed on a MAT-Finnigan 262 RPQ-plus multicollector mass spectrometer (Finnigan Corp., San Jose, CA) at the Vrije Universiteit Amsterdam using a static routine. The isotope ratios were corrected for mass-fractionation to ^86^Sr/^88^Sr = 0.1194. All measurements were referenced to the NBS987 standard, which gave a mean ^87^Sr/^86^Sr value of 0.710241 (*n* = 10) over the period of the study. The samples were run to an internal precision of ± 0.000006 (1SE) or better. The total procedural blanks (*n* = 5) provided a negligible contribution (mean 65 pg). All statistical assessments were performed in SPSS 22.0 (IBM SPSS Statistics for Macintosh, Armonk, IBM Corp.).

## Results

**Fig 3:**
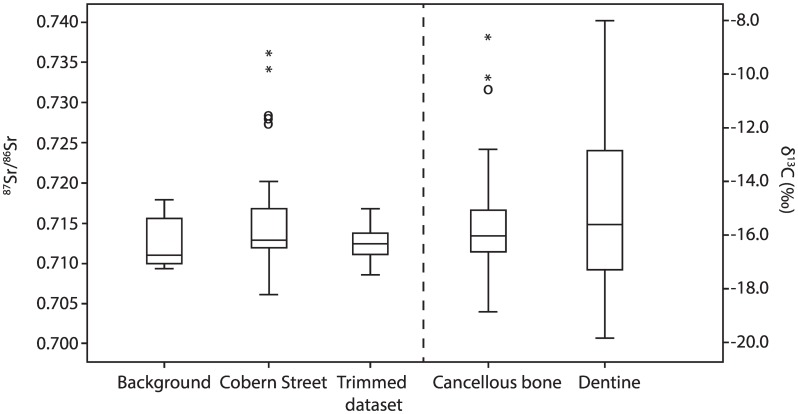
Tukey’s schematic boxplot showing ^87^Sr/^86^Sr variation for the background data (*n* = 18: Sealy *et al*. [69] and Balasse *et al*. [68]), the (trimmed) Cobern Street data (this study), and the variation in δ^13^C for the dentine (*n* = 36) and cancellous bone (*n* = 50) samples from Cox *et al*. [21]. Key: the boxes represent the interquartile range (IQR: Q3-Q1), the central line indicates the median. The whiskers represent Q1–1.5*IQR and Q3 + 1.5*IQR. The circles represent mild outliers (>1.5*IQR), the asterisks extreme outliers (>3*IQR). The trimmed dataset resembles a normally distributed dataset in which the statistical outliers are excluded.

Strontium, carbon and nitrogen isotope ratios from 35 individuals are reported in Table 3. Knowledge of the local isotopic background signature is essential for accurate interpretation of the isotopic data. Available human δ^13^C isotope values from Cox *et al*. were therefore reassessed to gain (additional) insight into the carbon isotope range specific to the Cape non-European underclass. Intra-population comparisons of carbon isotopes seem to provide the best way to identify individuals as migrants based on a different isotopic composition compared to the majority [90]. Therefore, to calculate the range of non-European underclass δ^13^C values local to the Cape, we reanalysed all human δ^13^C data from Cox *et al*. [21]. A statistical assessment of the carbon isotope data showed that the variance in δ^13^C_dentine_ was three times as high as the variance in δ^13^C_cancellous_ (11.3 and 3.6 respectively), indicating more diverse childhood diets, which converged in later life to a narrower range. We interpret these data as indicative of the Cape diet of this group (Table 4). The data are presented in a Tukey’s schematic boxplot in Fig 3 to display the variations in δ^13^C of the dentine and cancellous bone samples. By interpreting the mild outliers and extreme outliers in δ^13^C_cancellous_ (indicative of diet in the last few months to years of life) as non-local to the Cape, a local dietary δ^13^C range between -18.8‰ to -13.5‰ can be inferred (Fig 3). This range corresponds to a diet consisting of predominantly C3 foods with an approximate 25% to 65% contribution from C4 and/or marine food resources (see Fig 6 in [91]).

**Table 3:** Osteological, carbon, nitrogen, and strontium isotope data from 35 individuals from the Cobern Street informal burial site, Cape Town, dating to the colonial period.

Burial type	Burial #	UCT #	Age (yr)	Sex	δ^13^C dentine (‰)	δ^13^C cancellous (‰)	Δδ^13^C (‰)	δ^15^N dentine (‰)	δ^15^N cancellous (‰)	Δδ^15^N (‰)	Element #	^87^Sr/^86^Sr	± 2 S.E.
B	3	460	20	M	-16.4	-16.5	0.1	11.8	11.1	0.7	16	0.71375	0.00001
B	4	458.1	17–18	F		-18.8	-		12.7		16	0.71581	0.00001
B	10	498	35–40	F	-16.6	-15.9	0.7	13.3	12.9	0.4	26	0.71195	0.00001
B	12	500	35–40	M	-17.1	-16.4	0.7	11.2	12.4	1.2	46	0.70862	0.00001
B	13	501	30–40	M	-16.3	-16.2	0.1	12.4	13.2	0.8	46	0.71350	0.00001
B	14	502	45–55	F	-19.2	-16.9	2.3	8.8	8.7	0.1	46	0.71274	0.00001
											47	0.71336	0.00001
											48	0.71234	0.00001
B	15	504	25	M	-17.4	-16.6	0.8	10.9	12.4	1.5	46	0.71328	0.00001
B	18	508	>50	F	-13.9	-16.2	2.3	11.8	12.6	0.8	26	0.71465	0.00001
											47	0.71499	0.00001
											18	0.71167	0.00001
B	20A	510 +	25–30	M	-13.8	-12.8	1.0	9.8	9.6	0.2	16	0.72738	0.00001
											17	0.72858	0.00001
											18	0.72278	0.00001
B	20B	511 +	16	F	-12.2	-8.6	3.6	7.5	7.5	0.0	46	0.71675	0.00001
											47	0.71522	0.00001
											38	0.71570	0.00001
B	20C	512	1,5–2	?	-16.2	-16.2	0.0	16.1	14.1	2.0	71/2 or 81/2	0.71194	0.00001
B	21	514	25–35	F	-11.3	-16.0	4.7	12.3	12.3	0.0	46	0.71219	0.00001
											47	0.71158	0.00001
											38	0.71047	0.00001
B	23	516	17–19	F	-15.3	-16.6	1.3	12.0	13.4	1.4	36	0.71211	0.00001
											37	0.71109	0.00001
											38	0.71199	0.00001
B	27B	521	40–15	M	-16.4	-16.7	0.3	10.3	9.9	0.4	26	0.71900	0.00001
B	28	522	50	F		-13.9	-		11.9		46	0.71267	0.00001
B	34	528	14–15	F	-19.8	-18.8	1.0	10.3	9.9	0.4	46	0.71555	0.00001
B	40	535 +	12	F	-9.9	-10.5	0.6	6.1	7.3	1.2	46	0.72803	0.00001
B	41	536	35–50	M	-15.3	-16.2	0.9	12.1	10.8	1.3	36	0.71822	0.00001
B	44	542	40–50	F	-13.4	-14.1	0.7	14.3	11.7	2.6	46	0.73407	0.00001
											47	0.74186	0.00001
											48	0.71913	0.00001
B	45	543	50+	M	-16.7	-17.6	0.9	13.1	12.1	1.0	37	0.71343	0.00001
B	46	544	35–50	F	-17.7	-16.8	0.9	11.3	11.9	0.6	16	0.72015	0.00001
B	47	545	30–40	M	-15.3	-16.7	1.4	12.1	13.4	1.3	46	0.71260	0.00001
B	49	547	30–35	M	-10.3	-14.1	3.8	8.1	11.2	3.1	26	0.72830	0.00001
											47	0.74143	0.00001
B	50	548 +	35–50	M	-9.8	-16.0	6.2	7.6	12.6	5.0	46	0.70639	0.00001
											47	0.70603	0.00001
											48	0.70907	0.00001
B	51	549	35–40	M		-16.0	-		12.8		26	0.71017	0.00001
B?	52	550 +	25–35	F	-12.0	-10.1	1.9	7.2	8.6	1.4	46	0.70921	0.00001
											47	0.70938	0.00001
											48	0.71545	0.00001
B	54	552	30–35	M	-18.6	-15.8	2.8	9.9	12.3	2.4	46	0.71027	0.00001
											47	0.71044	0.00001
											48	0.71221	0.00001
B	56	554	35	M		-16.5	-		11.2		46	0.71387	0.00001
B	58	556	35–40	F	-11.9	-14.2	2.3	11.7	12.0	0.3	32	0.71233	0.00001
											17	0.71182	0.00001
											48	0.71378	0.00001
B	60	558 +	30	F	-8.0	-15.3	7.3	7.2	12.8	5.6	36	0.73605	0.00001
											37	0.73566	0.00001
											1/28	0.73608	0.00001
B	61	559	35	M	-15.5	-15.2	0.3	14.6	14.0	0.6	46	0.71183	0.00001
C	32	526	50–60	M	-18.0	-15.9	2.1	11.4	14.1	2.7	46	0.71225	0.00001
											17	0.71093	0.00001
											48	0.70923	0.00001
C	57	555	20–30	F	-18.4	-15.6	2.8	11.8	13.7	1.9	36	0.71011	0.00001
											47	0.71023	0.00001
											18	0.70947	0.00001
C	59	557	40	M	-17.6	-17.1	0.5	8.1	12.2	4.1	36	0.70600	0.00001
											37	0.70618	0.00001
											18	0.70602	0.00001
C	65	563	22–25	F	-15.6	-14.9	0.7	10.8	13.3	2.5	26	0.70921	0.00001
											27	0.70921	0.00001
											18	0.70910	0.00001

Key: Initial osteological investigations are executed and published by the Western Cape Physical Anthropology Group (see [71] for details). Carbon and nitrogen isotope data from Cox *et al*. [21]. Intra-individual differences between δ^13^C_dentine_ and δ^13^C_cancellous_, and δ^15^N_dentine_ and δ^15^N_cancellous_ are expressed as Δδ^13^C and Δδ^15^N respectively. Burial type refers to supine/Christian style (B) or facing Signal Hill/Muslim style (C). UCT number refers to the accession number. “+” indicates the presence of intentional dental modifications (see Table 1 for details). Dental element notation conforms to *Fédération Dentaire Internationale* (details presented in Table 2).

**Table 4:** Statistical assessment of human ^87^Sr/^86^Sr data (this study) and δ^13^C [21] data from Cobern Street, Cape Town.

Statistics	^87^Sr/^86^Sr	Trimmed ^87^Sr/^86^Sr	δ^13^C_dentine_	δ^13^C_cancellous_
N	33	23	36	50
Mean	0.71527	0.71239	-14.98	-15.54
Median	0.71274	0.71236	-16.25	-16.20
Standard deviation (1σ)	0.00696	0.00215	33.63	19.07
Standard deviation (2σ)	0.01393	0.00431	67.26	38.14
Variance	0.00001	0.00001	11.31	3.64
Minimum	0.70600	0.70862	-19.80	-18.80
Maximum	0.73605	0.71675	-5.30	-8.60
Range	0.03006	0.00813	14.50	10.20

Key: The trimmed dataset resembles a normally distributed dataset in which the statistical outliers (*n* = 10) are excluded.

The ^87^Sr/^86^Sr of the human enamel data range from 0.70600 to 0.73605 with a mean of 0.71527 ± 0.015 (2σ). The extremely wide range in strontium values with no apparent clustering of values indicated that the individuals in this study came from very diverse geological regions. A nonparametric Mann-Whitney U test was performed to quantify differences between males (*n* = 17) and females (*n* = 17). The results showed no statistically significant difference between the median ^87^Sr/^86^Sr ratios (U = 122, z = -0.775, p = 0.438) of males (0.714051 ± 0.00621) and females (0.716680 ± 0.00827).

Insight into the local strontium signal is gleaned from a statistical assessment of the first molar and deciduous teeth data from Cobern Street (*n* = 33). As in Wright [92], it is assumed that the ^87^Sr/^86^Sr ratios of a ‘local’ population are normally distributed. A Shapiro-Wilk test rejects the hypothesis that the Cobern Street dataset resembles a normal distribution (*W* = 830, df = 33, *α* = 0.000). Exclusion of statistical outliers (*n* = 10) results in a normally distributed dataset (*W* = 0.943, df = 23, *α* = 0.677) in which the mean and median coincide (0.71239 and 0.71236 respectively). Based on the human data, an approximate local range of 0.7086 to 0.7167 could be defined which was subject to later refinement (See section on Background ^87^Sr/^86^Sr data).

## Discussion

### Background ^87^Sr/^86^Sr data

A large amount of regional ^87^Sr/^86^Sr data, both biological and geological, are available for the Greater Cape Floral Region and surrounding areas of South Africa (e.g., see [67] for an extensive overview). These latter data are, however, not incorporated in this study due to A) the diverse nature of the sample types (biosphere/biological and geological); B) the distance of the samples location from Cape Town (up to ±150 kilometre); and/or C) the relatively low precision of the data due to choice of analytical method (±0.005, resulting in a large data range from similar sample types: Δ^87^Sr/^86^Sr_MIN-MAX_ = 0.023). Consequently, as explained in the section on determination of geological origins through isotope analysis, the regional strontium values were estimated solely on faunal samples. Local modern and archaeological faunal strontium data from wild and domestic animals from the south-western Cape within a 150 kilometre radius of Cape Town were reported by Balasse *et al*. [68] (*n* = 8) and Sealy *et al*. [69] (*n* = 10). Sealy and colleagues [69] found that humans and animals living on the Holocene coastal sands exhibited ^87^Sr/^86^Sr ratios between 0.7094 and 0.7117. The strontium ratios of modern fauna found in the (Pre-) Cambrian inland resource zones are characterised by higher ratios ranging between 0.7154 and 0.7179. The Cape granite areas surrounding Cape Town are characterized by bioavailable ^87^Sr/^86^Sr ratios ranging between 0.7099 and 0.7107 [68]. Based on the available modern and archaeological faunal data there appears to be an isotopic compositional gap in the local environment between 0.7117 and 0.7154 (Fig 2).

The human ^87^Sr/^87^Sr local signature was found to range from 0.7086 to 0.7167, simultaneously overlapping with the Sealy *et al*. [69] and Balasse *et al*. [68] datasets and filling the compositional gap left by the faunal data. Thus, in this study, a conservative local ^87^Sr/^86^Sr range, 0.7086 (human data) to 0.7179 (Pre-Cambrian bioavailable data), is used for the Cape (Fig 2). This range is remarkably wide due to the geological diversity of the region and hence not especially diagnostic. Moreover, the range will probably encompass the vast majority of the human data. As a result, the extent of human mobility is likely to be significantly underestimated, and the number of individuals assigned as non-local to the Western Cape should be considered a minimum.

### Human isotopic data

Based on the strontium data, a minimum of ten individuals were found to have not been born at the Cape, three of whom were female (burials 44, 46 and 60), while six were male (burials 20A, 27B, 41, 49, 50 and 59) and one was a child (burial 40). It seems reasonable to speculate that their migrations might have coincided with enslavement or change of ownership. Despite the absence of significant Δδ^13^C and/or Δδ^15^N in four of the assumed local or second- or subsequent generation slaves (burials 27B, 40, 41 and 46, see Table 1 and [21]), these individuals appeared to be alien to the Western Cape based on their ^87^Sr/^86^Sr ratio. It was therefore concluded that the isotopic difference between δ^13^C_dentine_ and δ^13^C_cancellous_ and/or δ^15^N_dentine_ and δ^15^N_cancellous_ did not provide reliable evidence for the presence of migrants. The non-local male dataset was isotopically more varied; its standard deviation (0.0089) was almost 1.5 times as high as the non-local female dataset (0.0061). Although the sample sets are small, this difference may indicate a larger variety in geological origins of the male population.

**Fig 4:**
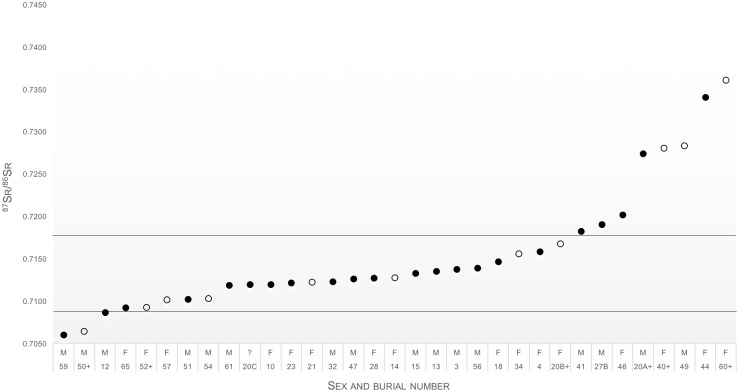
Strontium isotope data from M1 and deciduous teeth data from 33 individuals. The horizontal lines mark the local strontium range. Key: “+” next to sample number indicates the presence of intentional dental modifications. Open symbols identify individuals who exhibit non-local δ^13^C_dentine_ values. The standard errors in the analytical data are smaller than the plotted symbols.

**Fig 5:**
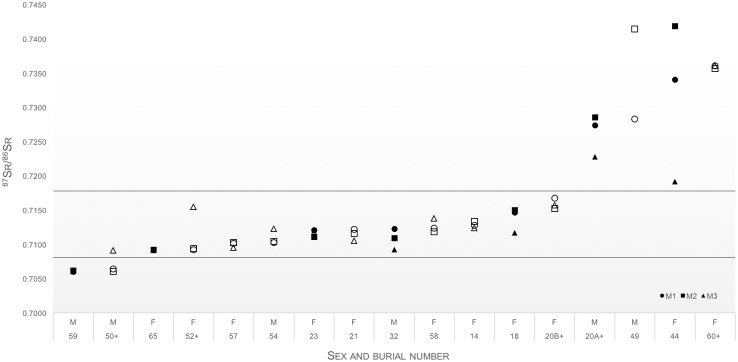
Multi-dental elemental strontium isotope data from 17 individuals. The horizontal lines mark the local strontium range. Key: “+” next to sample number indicates the presence of intentional dental modifications. Open symbols identify individuals who exhibit non-local δ^13^C_dentine_ values. The standard errors in the analytical data are smaller than the plotted symbols.

Figs 4 and 5 display the first molar and deciduous teeth ^87^Sr/^86^Sr data (*n* = 33) and multi-dental elemental ^87^Sr/^86^Sr data (*n* = 17) respectively. The data range from 0.7060 to 0.7419. The majority of these individuals (*n* = 22) also displayed a local childhood dietary carbon isotope signal (δ^13^C_dentine_: -18.8 to -13.5‰). Only four individuals, two males, one female and one child, exhibited both non-local strontium and carbon isotope values (burials 40, 49, 50 and 60). Burial 40, a child aged 12, had a significantly different diet during life (δ^13^C_dentine_: -9.9‰; δ^13^C_cancellous_: -10.5‰), which was dominated by C4 food resources [21]. In addition, this individual displayed a type of intentional dental modification characteristic of people of Mozambican descent [20, 21, 93]. This individual’s elevated ^87^Sr/^86^Sr ratio, 0.72803, was consistent with provenance from the radiogenic Phanerozoic and Precambrian bedrocks in Mozambique, further supporting possible Mozambican origins. Seven individuals, burials 14, 20B, 21, 34, 52, 54 and 57, exhibit local ^87^Sr/^86^Sr ratios, but their δ^13^C_dentine_ values deviated from the local range. They may therefore also be assigned as non-local.

All five individuals who exhibited culturally modified teeth (burials 20A, 20B, 50, 52 and 60; all type B burials) were found to be of non-local descent based on either ^87^Sr/^86^Sr (20A) or δ^13^C_dentine_ (20B and 52), or both proxies (50 and 60). These results verify the earlier assumption that the use of only one biogeochemical proxy may lead to the underestimation of the number of non-local individuals. The fact that all individuals with intentional dental modifications exhibited non-local ^87^Sr/^86^Sr and/or δ^13^C_dentine_ values was expected. It has been suggested that imported slaves quickly abandoned this practice to avoid recognition if they ran away [72, 94]. It would be unlikely that locally born individuals would continue this cultural practice. Based on the multi-dental elemental strontium data, individuals 20A and 60 lived in more radiogenic areas than the Cape in early life. Individual 50 originated from a geological area with less radiogenic strontium. Individual 20A appears to have experienced at least two migration events: one at the age of circa 8 (Δ^87^Sr/^86^Sr_M1-M2_ = 0.0012), and one after 16 (Δ^87^Sr/^86^Sr_M2-M3_ = 0.0058). In contrast, the isotopic data of individual 60 indicates residential stability until the age of at least 16 (Δ^87^Sr/^86^Sr < 0.001). The strontium isotope data for individuals 20B and 52 were compatible with the local range of strontium isotope ratios. The presence of dental modifications and a predominantly C4 based childhood diet (δ^13^C_dentine_ ~-12‰), however, precluded these individuals from being locally born. Burials 20A (a male between 25 and 30 years at time of death) and 20B (a young female of approximately 16 years of age) were buried together with a 1.5–2-year-old child (20C) in a common shaft and shared a single coffin [71]. The isotopic data and the presence of dental modifications showed that individuals 20A and 20B were of non-local descent, but did not share common geographical origins. Perhaps their dietary shift towards a more C4 signal, implied by the change in δ^13^C between dentine and cancellous bone, corresponded to their transportation to a central slave market from where they were bought and subsequently shipped to the Cape. They died shortly after arrival, before their bone was able to remodel and incorporate the local dietary isotopic signature. The ^87^Sr/^86^Sr ratio and δ^13^C_dentine_ value of the child, however, are consistent with the inferred local values.

The majority of the 12 individuals without dental modifications for whom multi-dental elemental analysis was performed exhibited strontium ratios compatible with the Cape indicating possible local provenance (*n* = 9). The carbon isotope data for individuals 18, 21 and 58, however, indicated a strong reliance on C4 food resources uncharacteristic of the Cape diet (all δ^13^C_dentine_ >-13.5‰). Thus despite strontium isotope ratios compatible with the estimated local range, local origins were unlikely. A similar conclusion was drawn for individuals 14, 54 and 57: their childhood diet consisted of C3 foods, but in much larger proportions than the inferred local diet at the Cape. Hence, the use of strontium isotope ratios alone as a diagnostic tool at the Cape will invariably lead to an underestimation of migrants. As a result, just four out of the seventeen individuals selected for multi-dental elemental sampling exhibited both δ^13^C_dentine_ values and ^87^Sr/^86^Sr ratios compatible with the inferred local ranges. From the available data, it was not possible to determine if these individuals were second or subsequent generation slaves, or had consumed a comparable diet to that of the Cape and hailed from a geologically similar region.

In addition to individual 20A, multiple migration events were also evident from the strontium isotope data of individual 44. This 40 to 50-year-old female was transhipped to more radiogenic regions after the age of 3 (Δ^87^Sr/^86^Sr_M1-M2_ = 0.0078) and experienced another (forced) migration after the age of 7 (Δ^87^Sr/^86^Sr_M2-M3_ = 0.023). As with individual 44, individual 49, a 30 to 35-year-old male, exhibited an M2 ^87^Sr/^86^Sr ratio that was significantly more radiogenic than his M1 ratio (Δ^87^Sr/^86^Sr_M1-M2_ = 0.013). Due to the absence of the 3^rd^ molar, it could not be established whether this individual’s migration to the Cape occurred after the age of 7 or 16.

Individual 59 plotted outside of the Cape strontium range throughout life. The ^87^Sr/^86^Sr ratios for this individual were similar to those of the M1 and M2 of individual 50, however, their differing childhood dietary habits (δ^13^C_dentine_ -17.6‰ and -12.0‰ respectively) suggested different geographical origins for the two males.

Individuals 32 and 59 (males) and 57 and 65 (females) were buried facing Signal Hill (type C burials, see Table 3), a sacred place for Cape Muslims. Due to their orientation, it was deduced that these individuals were Muslims. During the period under investigation, Islam was present in all corners of the Indian Ocean basin, including the Cape where slaves favoured it to Christianity for its inclusiveness [95, 96]. Unsurprisingly individuals from burial type C showed a wide range of Sr isotope ratios, ranging from 0.70600 to 0.71225, implying origins from very different geological regions. Based on combined carbon and strontium isotope data, two individuals (57 and 59) were undoubtedly of non-local descent. Individual 59’s low ^87^Sr/^86^Sr_M1_ ratio (0.70600) allows a tentative assignment to a region characterised by a young volcanic geology, such as the Indonesian archipelago, the Deccan traps region of India or volcanic islands in the Indian Ocean.

## Conclusion

We demonstrated the utility of relating dietary isotope data to strontium isotope data to enable a more accurate identification of individuals as local or non-local. The approach is particularly useful for migrations in which the geographical relocation is associated with the adaptation to new dietary habits.

The variable geology and the current absence of a comprehensive biological or biosphere database from the region in the near proximity of Cape Town, however, prevents the accurate delineation of the local strontium signal for the Cape Town region. As a result, the relatively broad local ^87^Sr/^86^Sr signature undoubtedly led to an underestimation of the number of non-local individuals. We argue, however, that this outcome is preferable to the overestimation of migrants. The use of only one isotopic proxy for migration (either strontium or carbon) generates an incomplete and inaccurate number of non-locally born individuals. This study demonstrates the efficacy of using multiple lines of evidence to generate a more reliable assessment of migration.

We conclude that the absence or the presence of a significant dietary shift cannot be used alone as a reliable proxy for migration. In contrast, a δ^13^C_dentine_ value that deviates from the assumed local range seems to identify migrants remarkably well and is as indicative as the presence of intentional dental morphological modifications, a practice not reported at the Cape. As a result, based on the combined interpretation of the osteological, carbon isotope and strontium isotope data, a minimum of 54.5% of the investigated population can be identified as non-local to the Cape (*18/35*).

Further identification of non-locally born individuals might be feasible using additional isotopic proxies such as lead (^206/207/208^Pb/^204^Pb and ^207/208^Pb/^206^Pb) and oxygen (δ^18^O). Lead isotope analysis was not undertaken in this study, however, as it requires a minimum of 120 mg of enamel powder for archaeological samples, destroying most of the dental element. Recent analytical developments, however, now offer this possibility as sub nanogram amounts of Pb can now be analysed [97]. Although oxygen isotope analysis may give additional information, expected oxygen isotope values in coastal regions of South Africa and countries bordering the Indian Ocean basin partly overlap, hampering an accurate interpretation of a person’s geological provenance (see e.g. Fig 3 in [98]).

This is the first extensive isotopic study that elucidates the complexity of the multi-directional Indian Ocean slave trade and sheds light on possible provenances. To date, the history of the Indian Ocean slave trades after European involvement has been highly dependent on the historical record in which subaltern populations are not always well represented. Future interdisciplinary research (combining isotopic data with palaeogenetics) of non-Europeans at the Cape will offer a direct route to assess a neglected history.
